# Sensor platform for assessment of water usage patterns in informal settlements

**DOI:** 10.1038/s41598-023-46236-3

**Published:** 2023-11-02

**Authors:** Andres Rico, Kent Larson, Mayra Gamboa

**Affiliations:** 1 Media Lab, Massachusetts Institute of Technology, Cambridge, 02139 USA; 2 CS Lab, Universidad de Guadalajara, 45180 Guadalajara, Mexico

**Keywords:** Engineering, Environmental social sciences, Sustainability

## Abstract

Rapid urbanization has intensified pressures on global water systems, particularly impacting informal settlements. Understanding water usage patterns within these settlements is of importance for better addressing water scarcity issues. Current methods for gaining information about water within these settings tend to lack spatio-temporal granularity and miss complex patterns of behavior related to water usage. As a consequence, there is a shortage of the reliable quantitative measurements needed to improve water management processes and modeling. Here we introduce a low-cost sensing platform for water assessment in informal settlements. Households within these types of settlements, lacking water utility connections and piping, often use storage tanks and buckets to distribute, store, and consume water; hence, the platform consists of four distinct sensor modules that can be placed on these types of water infrastructure. Evaluated in controlled settings, the sensors prove to be reliable for measuring water quantity, quality, and usage. Field testing within an informal community in Mexico reveals that the system can comprehensively track multiple tank storage levels, assess water quality, and capture bucket usage patterns without disrupting a household’s common activities or infrastructure. Our validation shows the technique’s potential to improve water management in informal communities, while opening opportunities for enhancement of water-related research and policy making through combinations of top-down and bottom-up interventions.

## Main

Rapid urbanization and climate-related challenges are expected to put great pressures on global water systems^1^. Cities are projected to concentrate 58% of the world’s population over the upcoming five decades^2^. Within this scenario, simulations estimate that almost half of the world’s population will face water scarcity by 2050^1^. Further aggravating the problem, city land area is expected to grow 141% in low-income countries and 34% in high-income countries^2^, leading to accelerated growth rates of informal settlements^3^. As rapid urbanization exacerbates regional water scarcity^4,5^ and most urban growth will be in low-income informal settlements^2^, understanding water usage patterns within these types of settlements is of substantial importance for better addressing global water scarcity issues^1,6,7^. Research points to novel data-based water management processes and techniques as crucial mechanisms for managing scarcity and securing resource accessibility^1,6,8,9^. For example, a holistic understanding of a city’s water sources, consumption patterns, distribution systems and water quality levels can improve water management policies and practices, in both planned and informal urbanization^6^.

In regions with planned urbanization, water management and monitoring is carried out through a range of methods and scales^10^. For example, satellite enabled remote sensing can be used to estimate water reserves’ availability and quality^11,12^; consumption and quality statistics can also be sourced from meters and stations that belong to private and public water infrastructure providers^13,14^; sensors and machine learning can be used for detection of leaks in distribution networks and water loss estimation^15^, and specific household and community usage can be monitored through smart flow sensors placed in a household’s pipe infrastructure, supply meters or through machine learning algorithms and statistical behavioral or flow analysis^16–18^. In contrast, how water is sourced, stored and used in informal settlements creates a unique set of challenges that make it difficult to have access to up-to-date, granular and timely information describing water at the community, household or individual scales^19^.

Absence of formal water infrastructure makes it hard to setup conventional meters or fixed stations to collect data describing community water usage and water quality. Lack of formal piping infrastructure also leads communities to rely on varying mechanisms to source enough water to meet their basic daily needs^20–23^. For example, households capture rain water, access it through community wells, have water delivered by trucks, clandestinely connect into formal infrastructure lines, have water delivered by governmental programs or access it via unregulated wells^24^. Different means of collection means that water quality will vary depending on the source of the water^25^. Another important challenge for mapping water usage is that sourcing is highly seasonal and context dependent^26^. For example, in some areas, rain water is a viable option only in wet seasons. During dry periods, water trucks and wells might tend to be frequented more often. This means that the sourcing mix within a given household is constantly evolving^20^. Once water is obtained, due to uncertainty of sourcing, households typically use multiple containers with different capacities, shapes, materials, and locations to store water within homes^20,27^. Diversity in materials used to make tanks and varying container dimensions also affect water quality evolution during storage periods^28^. Additionally, multiple storage containers make it hard for households to constantly estimate the total amount of water that they have available to them^29^. This might be attributed to tanks being spread out around homes and terrain, sometimes, in places that are difficult or dangerous to access such as underground or unstable house roofs. Finally, with lack of pipe infrastructure, usage of water is commonly carried out through buckets. Buckets are the main vehicle of transportation and use of water within homes and streets^30,31^. The use of buckets also makes it challenging to assess consumption patterns within households. For example, it is difficult to know how much of the water is used for drinking, cooking, sanitation, cleaning, or watering plants, amongst other activities.

Current efforts to gather information about water within informal settlements rely on collecting data through in-person interviews, surveys, government led census campaigns, site investigations and manual measurements^32,33^. While these instruments have had implementations around all continents and are able to get useful information, they are prone to human error; fail to track key variables that describe complexity of water usage, such as detailed daily consumption per household; many times, are outdated by the time they are published; and tend to be costly in terms of human labor and logistics^34,35^. Furthermore, as surveys happen as isolated yearly or multi year events (depending on country), they are not able to capture granular seasonal changes. This makes it challenging to know constant changes in water quality and what specific usage is given to the water that they store in their homes. Despite their drawbacks, surveys and interviews manage to give a good sense about the available water infrastructure and general practices within households^29^. They can help to access information about types of tanks, storage availability, and determining household infrastructure locations but these processes are far from providing the high quality quantitative measurements that are needed to improve hyper-local water management processes and modeling^36^. Water specific research highlights the need for robust quantitative data collection methods^20,29^. The stated limitations greatly compromise the richness of information that later informs households, researchers, infrastructure development, and water management practices^29^. It is clear that there is a need for systems that are able to collect granular information that can quantitatively complement currently employed methods.

Here, we report a low-cost, distributed sensing network for water management in informal settlements. The system is able to gather temporally granular information about water usage patterns, quality of water, and the amount of water available in a given household. The proposed network can be modularly adhered to an informal household’s water infrastructure, such as buckets and water storage tanks. As household dwellers do not need to actively engage with the system to collect data, the system is able to automatically gather information without disrupting a household’s common infrastructure usage and water handling practices. Subsequently, this sensing method can enable access to metrics that can describe weekly consumption, average daily consumption, correlations between water usage rates, container types and water quality, behavioral changes in dry periods and wet periods, as well as household water activity and usage patterns, amongst other variables that are currently not accessible through current state of the art practices and technologies. The use of low-cost, distributed sensing systems has the potential to improve water management practices in informal settlements in multiple ways. Firstly, it enables households to gain a better understanding of their water usage patterns. For example, these systems could promptly alert them when their water supply is running low, enabling them to manage their resources more efficiently. Additionally, they could help to optimize water usage based on variations in water quality. Secondly, by providing detailed and real-time information on water usage dynamics, this technology empowers decision-makers, researchers, and policymakers to develop customized interventions and strategies. These targeted approaches are crucial for understanding the unique needs and challenges faced by marginalized communities living in informal settlements.

## Results

### Low-cost, distributed sensing system architecture

**Figure 1:**
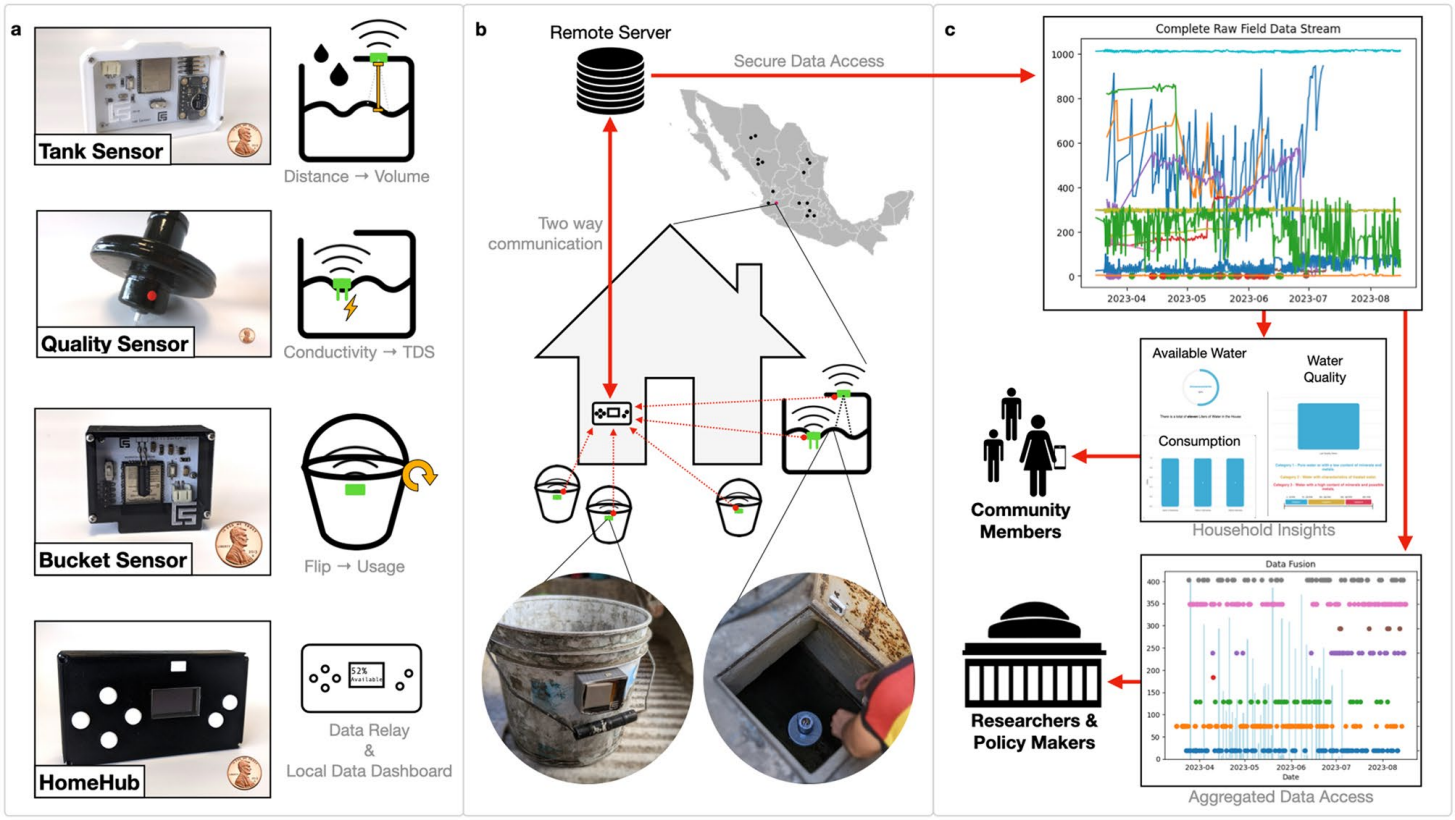
(**a**) Tank senor, quality sensor, bucket sensor and HomeHub prototypes. The tank sensor is used to measure water quantity in a container, quality sensor measures total dissolved solids (TDS), the bucket sensor measures frequency of usage of buckets around a house and the HomeHub device serves as a data relay and as a local user interface. (**b**) Field test for system installed in Mexico. We demonstrate multiple sensor types connected to a single HomeHub within a household. The architecture has the potential to allow for large scale informal settlement water resource monitoring. (**c**) Data can be accessed by communities through local interfaces or be used by governments, researchers and policy makers (through secure API’s) to improve water management models and infrastructure.

Our sensor network is made up of four different discrete units, one wired communication unit, *HomeHub* (see **a** in Figure 1), and three wireless battery-powered sensing units (quality sensor, tank sensor and bucket sensor). Sensing units connect to the HomeHub through low power radio and send data packets two times per day. The HomeHub receives information and uploads the data packets into a database for secure storage and later access through WiFi. As it is connected to the internet, the HomeHub device also queries the OpenWeather API^37^ and saves data describing current atmospheric and environmental conditions at the location in which it is installed. Importantly, the platform also uses the HomeHub’s screen to locally display relevant insights to users in the household. The local screen’s interface complements a web app (accessible on a phone, tablet or computer). The *quality sensor* (see **a** in Figure 1) is a floating buoy with an exposed electrode for measuring water conductivity. Water conductivity information is then used to estimate total dissolved solids (TDS). The *tank sensor* (see **a** in Figure 1) is placed on water storage container lids. It uses an optical distance sensor to measure the distance between the lid and the water line. The distance is then used to compute the current volume of water inside the container. Finally, the *bucket sensor* (see **a** in Figure 1) is a tilt switch sensor that closes and opens up a circuit every time a bucket gets flipped (either to be filled up or emptied). The device can monitor frequency of use for buckets around a household. The number of connected sensors is dependant on the amount of storage tanks and buckets that are used within a given household or experimental scenario. All of the system’s units use programmable, low-power, off-the-shelve components to enable scalability and replicability of the system. A complete bill of materials and circuit designs for each module can be found in supplementary materials for the publication.

### Sensor evaluations and characterizations

We first evaluate the system focusing on the comprehensive testing of its four units within controlled laboratory settings. Our evaluation encompasses an in-depth analysis of sensor accuracies, drift, and mean packet delivery errors. Through rigorous testing and characterization, we aim to provide a detailed assessment of the system’s performance, highlighting its strengths, shortcomings and the insights that it can enable.

**Figure 2:**
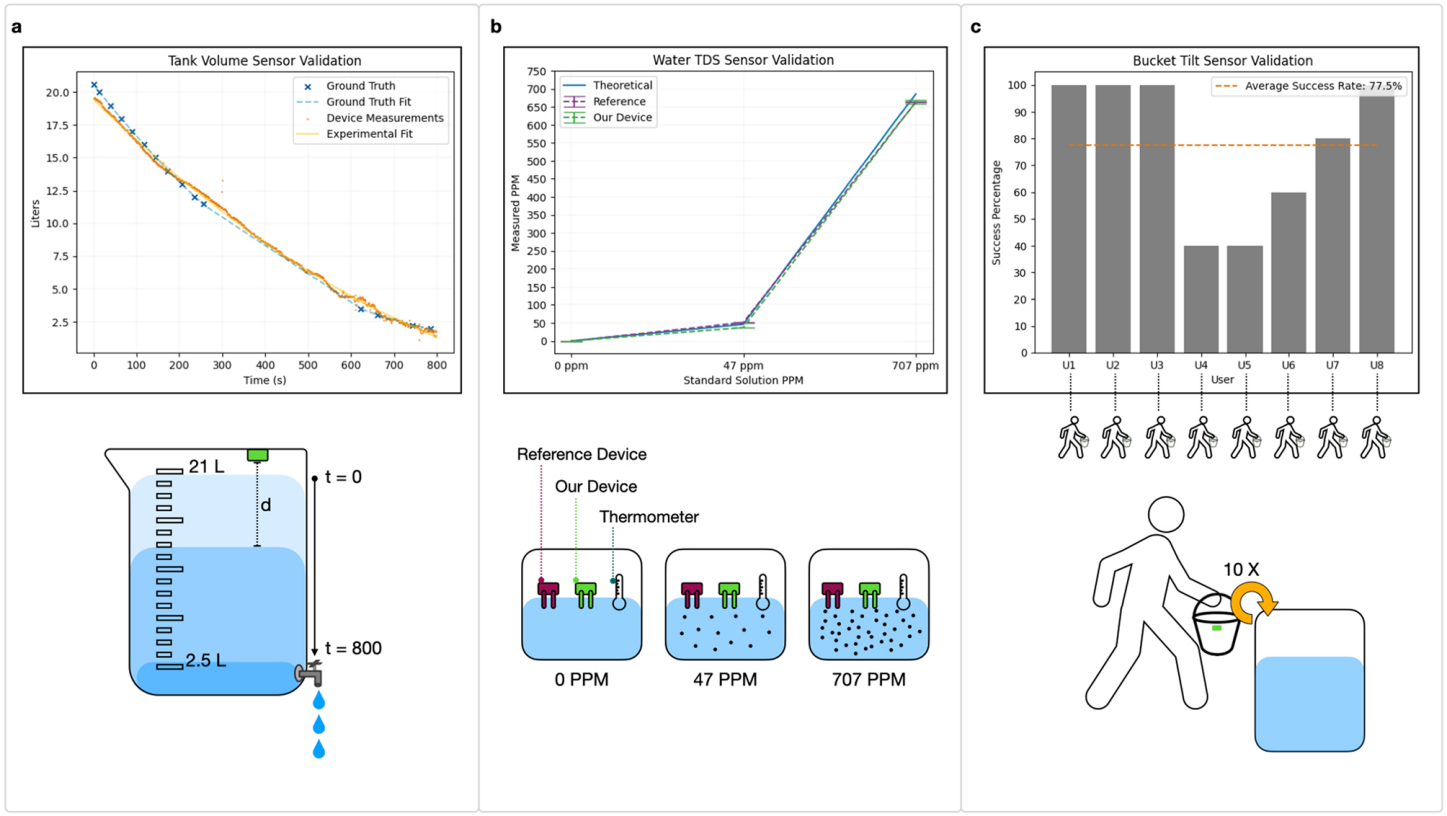
Sensor module evaluation plots and experimental setup diagrams. (**a**) Water volume experimental plots. The green plot shows the device volume calculations and the purple plot shows ground truth volume. To collect data, a tank sensor is mounted on the lid of a 21 L tank that is being drained at a constant rate. (**b**) Water quality sensor accuracy plots. PPM values were tested against a commercial device with standard solutions of 0 PPM, 47 PPM and 707 PPM. (**c**) Plots show success percentage for ten study subjects using a sensorized buckets. The average detection rate for all participants was of 77.5%.

We first evaluated the tank sensor’s performance when determining volume of water within a 21 L graduated container. We measured water volume in the container as water is being pumped out at a constant rate. Measurements were taken from the moment the container was full until it was empty. During testing, the tank sensor exhibited a mean error of $\pm 9.3\%$ when determining dynamic water volume, sampled at a frequency of 1.23*Hz* for 800 seconds (Reference **a** in Figure 2). The same device has a mean error of $\pm 2.3\%$ when determining distance between lid and a steady water line, sampled twice per day over a period of two months; using the same container as the one used for the dynamic experiments. Notably, error mean is reduced to $\pm 0.7\%$ when the sensor was employed to measure the distance between the lid and a steady solid wall, sampled twice per day for 5 months. Given that water usage within a household is typically not highly dynamic and measurements to a steady water line exhibit low average errors, we can ascertain that the sensor effectively tracks water volume within a container while maintaining an acceptable level of drift over extended time periods. Furthermore, the observed error rates of the device are considered acceptable, as the minimal differences would not be expected to have significant impacts on overall calculations and data patterns collected by the system. It is worth mentioning that the accuracy of water quantity measurements also depends on the precision of the container’s height and sectional area measurements, which are further discussed in the Methods section of the document.

Next, to validate accuracy of the water quality sensor, we compared readings from our device and a reference commercial TDS probe (Reference **b** in Figure 2). We used three standard solutions as our baseline to assess sensor accuracy (0 PPM, 47 PPM and 707 PPM). This evaluation helps us to understand the sensor’s performance at different known levels of dissolved solid matter as well as to estimate possible deviations from ground truth when sensors are deployed in the field. The reference commercial probe showed a mean error of $\pm 3.73\%$ compared to a mean error of $\pm 4.26\%$ for our device with 500 samples taken for each solution. This allows us to conclude that the device is operating within acceptable standards for estimating TDS concentrations within water containers. We note that our device contains an on-board temperature sensor that allows it to adjust its calculations to account for effects of temperature on water conductivity. Details about temperature compensation can be found in the Methods section.

Lastly, we evaluate the bucket sensor in its capability to capture key bucket usage states. In particular, the device is able to detect when a bucket has been flipped when in use. We assess that the sensor is able to detect such activity by asking eight study participants to fill up a bucket and empty it into a container (Reference **c** in Figure 2). This allows us to verify that the sensor is able to correctly capture the action of flipping a bucket independent of varying user motions. Test users are asked to repeat the sequence of filling up and emptying the bucket 10 times. The device showed a combined success rate of 77.5% for all of the experiment trials, of which half had success rates of 100% (see **c** of Figure 2). The user studies show that it is possible to encounter false positive and false negative bucket activations, expressed in low success rates for some users (See **c** in Figure 2). The user tests and field experiments allow us to assess that instances of false positives can arise when sensors indicate bucket tilting even when the bucket is not flipped. An example of this would be a bucket being left to rest upside down. Conversely, false negatives might occur when a bucket is flipped but the sensor does not register the tilt due to the usage being carried out in areas with weak connectivity or a user tilting it at an angle insufficient for the sensor to activate (as is the case for the majority of false negatives detected during user studies). With this, we can conclude that the device is able to timestamp instances of bucket use within a household with acceptable reliability. As each bucket has a unique ID, and location in a home, we can use the timestamps to measure activity patterns for different locations and uses of buckets around a given household.

**Figure 3:**
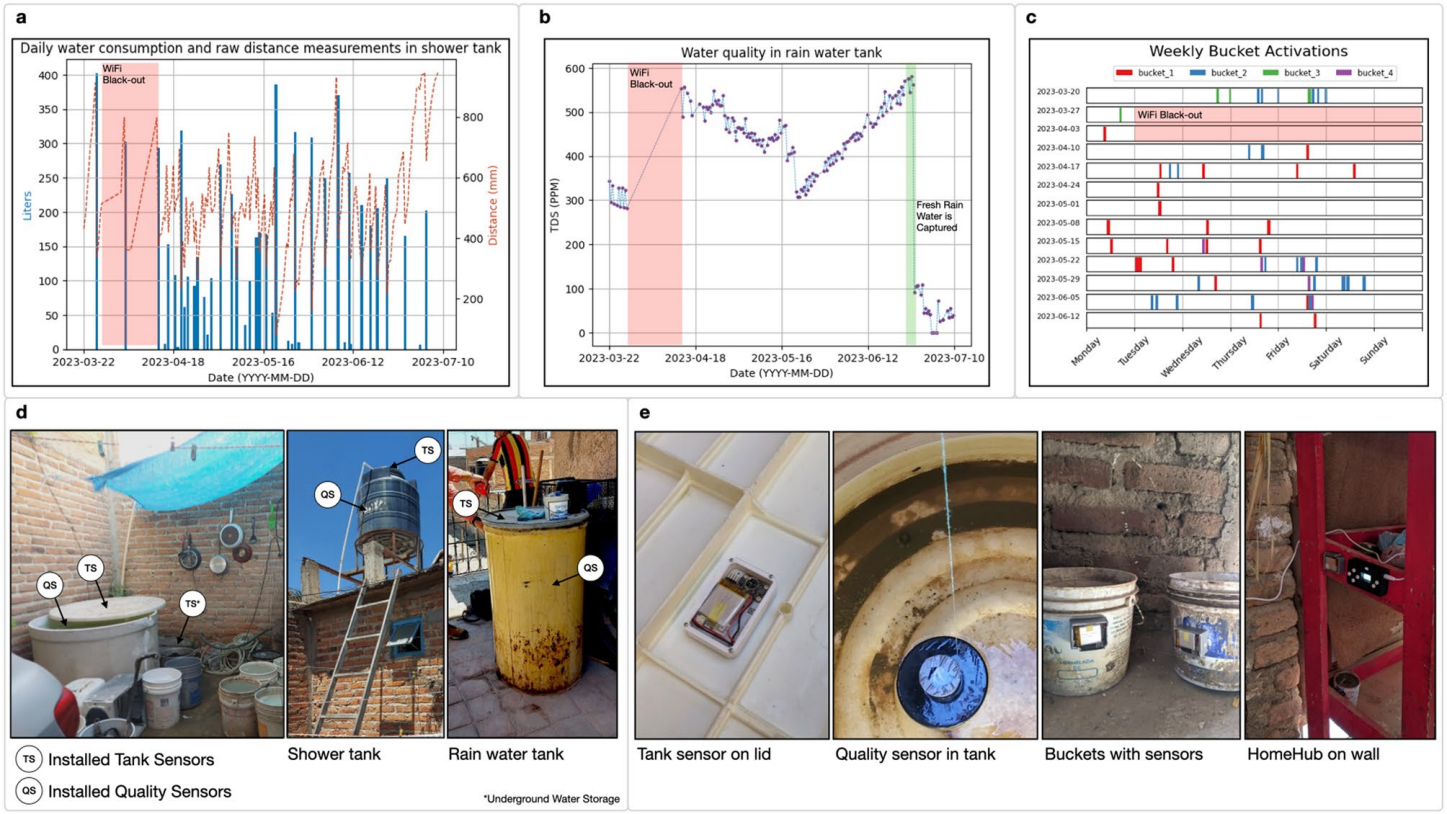
Field deployment sensor evaluations and installation images. (**a**) Water consumption plot for tank sensor placed in shower tank over field deployment period. (**b**) Water quality for quality sensor placed in rain water tank used for storage. (**c**) Activation plot with all bucket activations for the deployment period. (**d**) Sensor placement for quality and tank sensors in field deployment. (**e**) Images of installed devices around the household.

### Field deployment evaluation

We present results of a field evaluation of the sensors in a real-life deployment, specifically focusing on the platform’s suitability as low-cost sensing infrastructure for informal communities. Our evaluation encompasses verifying the system’s capability to granularly measure the water level in a tank, determine water quality within a storage container, and describe water usage within households through bucket activations. This includes assessing how the system is opportunistically embedded so that users do not need to change their common habits when interacting with water infrastructure within their homes.

For this experiment, we deployed a total of 12 devices in a household located in an informal settlement in the metropolitan area of Guadalajara, Mexico. The system collected data during 110 days. The installation comprised of one HomeHub, four bucket sensors, four tank sensors, and three quality buoys. Prior to the installation, we conducted an initial assessment of the household by conducting three interviews and two field visits with the household leader. In accordance with the value of surveys pointed out by literature above, this qualitative information provided insights into the common usage patterns of the infrastructure that were expected to be seen in the data. Through this process we were able to identify tanks that were used daily, tanks that were used for long term storage and typical location and usage, within the home, for each of the buckets. By integrating these qualitative details into our post-deployment data analysis, and through constant communication with the study participants, we were able to validate the sensor readings and identify any irregularities in the data more effectively. Please refer to the Methods section for more information on the interview process and field visits.

Humidity, dust and constant exposure to heat affect connectivity and performance of all the devices. Placement of the devices around the house also affects transmission capacity as data packets, in some cases, have to travel across dead connectivity zones. Therefore, we first evaluate the successful packet transmission rate of each node of the system excluding bucket sensor nodes. We do not consider bucket sensors in our success rate calculations as ground truth of activations for this type of sensor is unknown when deployed in the field. The platform, as a whole, showed an average successful packet rate for all tank and quality sensor devices of 65.03%. This packet rate includes lost packets from a time period with a WiFi black-out that lasted two weeks (shaded in red in the three sub figures of Figure 3) as well as lost packets from sensors that had manufacturing deficiencies or accidents in the field (e.g. whole submersion of devices in water). The success rate without considering the days with a WiFi blackout is of 71.9%. As a 50% success rate would mean that, on average, we are able to collect one data point per day per sensor, we can consider that the system’s data loss percentages are acceptable for being able to granularly map out the variables of interest within these types of households. The evaluated sensor types (tank and quality sensors) had at least one sensor with a success rate above 82.72%. This indicates that packet rates can be greatly increased if location of devices and detection of unconnected areas is optimized during installation. We use the data from sensors with the highest success rates of each sensor type to further evaluate specific capabilities of the system. This enables us to consistently evaluate various facets of the field data and derive further conclusions regarding the platform’s performance.

As it is not possible to have accurate ground truths within a 110 day in-field experiment, and we have already evaluated sensor accuracy in controlled laboratory settings, we evaluate sensor performance by empirically analyzing sensor information and corroborating it with qualitative information gathered through the interview process prior to installation. Figure 3 shows plots of data collected over the three month period for the tank sensor installed on the tank that is used for showering (successful data transference rate of 94.54%), the quality sensor placed in the storage container that is used to capture rain water and for long term water storage (successful data transference rate of 82.72%), and the bucket activations for all four bucket sensors. Field data shows that the tank sensor is able to adequately track the distance between the tanks lid and the water line showing daily oscillations (see Figure 2’s **a** plot.). We also show how these values can be converted and plotted as daily consumption of water for the respective tank. The tank sensor’s data shows the cyclical behavior that would be expected of a sensor mapping water quantity of a container that is regularly used by the household for showering. With this information we calculate an average daily consumption for the shower tank of 60.65 L. In comparison, data from tank sensors placed on long term storage containers shows a steady level of water on tanks and minimal consumption (likely due to evaporation). Quality of water within the rain capturing container shows that quality is in the range of 0 - 500 PPM (see Figure 2’s **b** plot). As expected, values change in time, due to evaporation, water being added or water being used. Drastic drops or changes coincide with moments in which large quantities of water were added or used. We can visualize a drastic drop that coincides with the filling of the container with freshly captured rain water (shaded in green). The bucket activation plot (Figure 2’s **c** plot) shows every time that a bucket is used across all weeks of deployment. Each color represents a different bucket. The data shows consistent cyclical behaviors and patterns of use that allow us to corroborate that the sensor is performing correctly and mapping household bucket activity usage as expected. Patterns of choice of days for using water around the house can be seen as well as days in which multiple buckets get used. Through interviews, we corroborate that Tuesdays and Fridays tend to be intensive water usage days as they are house chores days while Sundays have the lowest water use as household members use that day to rest. Interestingly patterns of use on Tuesdays and Fridays tend to defer from the ones seen on Wednesdays and Thursdays. This is due to different household members being responsible for chores around the house throughout the week.

Through qualitative and quantitative evaluations we can conclude that the deployment results indicate that the sensor system has reliable tank level tracking, monitors water quality within acceptable ranges, and is able to log detailed bucket usage patterns. This evaluation highlights the potential of the sensor platform to address water management challenges in informal communities, while suggesting opportunities for improvement in connectivity and device resilience. In challenging environments, equipping sensors with larger battery packs can increase reporting frequency and enhance daily coverage granularity. Despite the inherent challenges posed by the environment, the system’s achieved granularity surpasses the requirements for effectively mapping the variables of interest. Consequently, it represents a clear improvement and possesses clear complementary potential to currently employed methods.

## Discussion

Following, we discuss how the sensor platform’s data could be used alongside surveying methods and interviews to complement current water-related data availability within informal settlements. We also highlight some of the major limitations for the system, and ways in which they could be addressed in the future. As indicated by our field and laboratory results, the system is able to comprehensively track water quantity, quality and usage within households. This new data has the potential to enable more holistic data management models that address both planned and unplanned urbanization by complementing currently employed survey and interview methods. Furthermore, trends in various fields such as water management, development economics, urban planning, machine learning, and low-power sensing can address some of the limitations of this sensing technique or make effective use of the generated data to improve our current models and understanding of how water is sourced and consumed within these types of communities.

For community members, the system can offer valuable insights on optimizing water resource utilization. For example, by identifying water-intensive activities, households can allocate high-quality water for essential purposes while utilizing lower quality water for tasks that do not require as high a quality, such as cleaning external parts of the home or non-sanitary usages (flushing toilets). Achieving this local benefits would require significant improvement in the longevity of the sensors; challenged by technical aspects of the system as well as by social ones such as perceived benefit of the sensors (from community members), and theft. Maintenance requirements must also be addressed so that systems can be kept functional without being an extra burden for community members. If these challenges are addressed, the system could help individuals within the community to better understand their relationship with water, and aid them by improving local water management practices, specially in dry periods that might be more straining to households.

The platform can also enable the study of complex correlations between water infrastructure and other socio-economic indicators of a given community. For example, we could analyse how water quality varies by water source, the effects of different container types on water usage and quality over time, or examine poverty traps that may be exacerbated by water scarcity issues. Furthermore, correlations of consumption, weather patterns and water quality can give insights into the complexities that the typical water cycle in these communties can have; helping us to better understand forces and motivators that drive current consumption decisions as well as the impact that those decisions can have in a household’s quality of life.

Additionally, the use of these types of sensor networks, could lead to research interest in understanding how information systems and interfaces could be implemented to be most useful and minimally cognitively loading within the context of households in informal settlements. Increased adoption of these systems, by both communities and researchers, will be strongly dependent on the value that households perceive the information gives them. If households do not see continuous benefit from having the system within their homes, the potential of these collection alternatives will be attenuated. The design of novel information interfaces and the development of methodologies that allow households to build and install their own infrastructure in the future can be key for realizing the potential of the proposed data collection method. Device adoption could also be scaled by researchers in a more incremental manner through targeted short term sensor deployments. For example, specific households could be selected to run a temporary one month study with sensors instead of having the system in their homes for larger periods of time.

If deployed in multiple communities, the system has the potential to create a real-time and granular data set that is currently limited or unavailable. Further investigation is required to analyze the amount of houses that would be representative of a community as placing devices within all homes in a given community might not be feasible. Combining this new data set with existing survey, interview, and satellite data, could positively impact governance processes. As shown in this study, survey information can inform the design and installation of sensing infrastructure, it can highlight available infrastructure to be sensorized within a home and give information about common uses that each tank or bucket has. This information can then be complemented through qualitative information about the infrastructure such as the average daily consumption of water from a specific tank, the average quality that a given storage container has, and a detailed recollection of how buckets are used around a household. This combination of qualitative and quantitative methods could allow policymakers to have more up to date and granular information to integrate into their decision-making processes to improve resource allocation and better administer infrastructure upgrades. Moreover, aggregate data from the system can also inform the design of devices aimed at addressing water scarcity in these communities. For instance, by combining precipitation data with water usage data, it becomes possible to determine the dimensions of a rain capturing system that meets the real consumption requirements of the household. Optimal placement of the capturing system could then be decided through qualitative information describing house layouts along with observations of optimal places for avoiding theft of the system while maximizing rain exposure.

From the engineering perspective, advancements in energy harvesting and low-power sensing technologies could further enhance the capabilities of the presented platform by allowing for longer operation times for the sensors and further increasing sensor accuracies. Additionally, since connectivity and signal strength in informal settlements are not optimal and cause lost data packets and system malfunctions, large-scale deployment across different time periods can be utilized to gather data for training AI models that can estimate values for lost data packets, increasing the granularity, reliability, and accuracy of systems. Such models can also be adapted to estimate water consumption for different communities within the same region, even if they do not have access to the same sensing infrastructure.

In conclusion, the proposed system presents a promising approach to addressing critical water management challenges within informal settlements. The current implementation of the system shows a wide range of limitations as well; lost data packets, deployment times, device tampering, maintenance costs, and presentation of information to households, all need to be addressed in the future for these types of systems to become ubiquitous within settlements and within research activities. Ultimately, the integration of multidisciplinary research approaches will further enhance the capabilities and applications of this technique. Our present research highlights the system’s capability to comprehensively monitor water quantity, quality, and usage at the household level, providing valuable insights into water dynamics in unplanned urbanization scenarios. For community members, it offers actionable information for optimizing water resource allocation and enhancing water management practices. By integrating data from this system with other indicators, governance processes can be informed to guide infrastructure development and adaptive policies, ultimately contributing to more sustainable and water-aware urbanization patterns.

## Methods

### Networking architecture and wireless sensor node power management

The number of connected sensors is dependant on the amount of storage tanks and buckets that are used within a given household or experimental scenario, with a limit of 100 sensors per HomeHub. Sensor units locally communicate with the HomeHub device through *esp-now*, a low power Bluetooth low energy (BLE) communication protocol developed by Espressif^38^. The HomeHub is the only unit that is connected to the internet through WiFi. Everytime that the HomeHub receives a data packet form a sensor module, it securely relays the information to a remote database for storage and later access.

All of the devices are based on the ESP-32E micro controller and transciever^39^. We use the ESP32’s Deep Sleep capabilities to drastically lower power consumption of wireless sensor nodes. The tank and quality sensor use an internal timer to send a data packet to the HomeHub once every 12 hours. The sensing devices wake up, measure, connect with the HomeHub, send data and go back to deep sleep mode. The bucket sensor also uses the micro-controller’s deep sleep capabilities but instead of waking up every 12 hours, the tilt switches wake up the device through one of the device’s GPIO pins. Once awake, the device sends its ID to the HomeHub and goes back to deep sleep mode. Buckets that have regular use will wake up the device more often meaning that battery life is dependant on bucket usage.

### Distance to volume and estimation of water consumption

The water quantity sensor is placed on a tank’s lid. The sensor uses a VL53L4CX optical distance sensor^40^ to calculate the distance between the tank’s lid and the water surface. Distance is then used to estimate the volume of water contained within the storage tank. During installation, measurements of the tank are taken. Maximum internal height, width and depth are recorded and used to convert sensor readings into volumetric estimates. To calculate Volume at time *t* We use equation $V_{t} = (D_{max} - D_{t}) * A * \rho$ where $V_{t}$ is the total volume of water in the tank in Liters at time *t*, $D_{max}$ is the maximum height within the tank in millimeters (measurement with an empty tank), $D_{t}$ is the measured distance at time *t* in millimeters, *A* is the sectional area perpendicular to the distance measurement in square meters, and $\rho$ is water’s nominal density approximated at 1000 $kg/m^3$.

Water consumption is determined by analyzing the differences between data points over time. To calculate water consumption, we create an array called *V*, which contains the recorded volumes $[v_{t_1}, v_{t_2}, ..., v_{t_n}]$ at different time points. The range for *n* is defined by the initial and final dates of interest for the consumption period of interest noted as [*i* : *f*]. We define water consumption as occurring when the volume at a specific time *t* is smaller than the volume at the previous time point $t - 1$. Conversely, a larger volume at time *t* indicates water has been added to the container. By comparing all the data points to their preceding values within the specified range, we identify instances of water consumption. We sum up the absolute differences between consecutive data points that indicate consumption. This sum provides an estimate of the total water consumed during the given period. It is important to note that this method does not account for water consumption or addition that takes place between the sampling periods. To address this limitation, one could increase the sampling rate. However, doing so would come at the expense of battery performance, making it a costly trade-off. The 12 hour sampling window that we chose is set to balance this trade-off.

### TDS measurements and estimation of water quality

Total dissolved solids in water are calculated by reading the amount of ions diluted within a specific water body. The measurement of total dissolved solids (TDS) is commonly used when analyzing water samples to assess their quality and is considered relevant within many water quality indices^41^. As TDS is derived from water’s electrical conductivity (EC), TDS is an indicator of the presence of salts or other possible pollutants in water. While TDS can be greatly complemented with metrics such as turbidity, pH, dissolved oxygen, and bacterial content amongst others to create a complete water quality index^41^, measuring TDS is electronically simple, low-cost, and low power. These characteristics help us achieve crucial design constraints for our system. The measurements obtained by the system are therefore not complete for assessing true water quality but research shows that it can act as highly correlated indicator for it^42,43^.

Our design uses the Gravity Analog TDS amplifier circuit board and electrode^44^ connected to our custom PCB with a micro-controller unit that has an Analog to Digital Converter (ADC) with a reference voltage of 3.3*V*. We measure a raw voltage $V_{raw}$ between the leads of the electrode. As temperature has effects on the conductivity of water, we calculate a temperature correction coefficient, $\alpha$, through equation $\alpha = 1 + 0.02 * (T_{t} - 25)$ from^45^ where $T_t$ is the temperature at time of sampling *t* in degrees Celsius. The compensation is carried out through equation $V_{comp} = V_{raw} / \alpha$ where $V_{comp}$ is the compensated voltage. We then use the amplifier board’s manufacturer’s third order polynomial fit^44^ with shape $p(V_{comp}) = aV_{comp}^3 + bV_{comp}^2 + cV_{comp} + d$ with values $a = 133.42, b = -255.86, c = 857.39, d = 0$ to convert $V_{comp}$ into an EC measurement. EC and TDS have a strong correlation that is usually expressed by the equation $TDS = \beta * EC$^46^. Our implementation uses the standard value for water of $\beta = 0.5$.

### Household survey and interview

We carried out a series of interviews and field visits with the study household to inform the design and field evaluation of the devices. We made use of interviews that lasted around two hours each. Two of the interviews had an unstructured format, and one of them had a structured format. The reader can access all of the questions asked during the structured interview in the supplementary material for this publication.

During the structured interview, the participating household leader was able to describe, in general terms, their water infrastructure and common usage of that infrastructure. For example, they told us how many tanks they had within their household, which tanks they used for storing rain water or for storing water delivered by trucks, location of tanks around their house, what buckets were the ones they used the most, what were the typical weekdays for cleaning the house or for doing laundry, amongst other qualitative factors of this nature. These answers were used to plan the amount of sensors for each sensor type that would be deployed during the field study. e.g. As participants stated that they mainly used four buckets around the house, we installed sensors on those four buckets. Generally, participants were unable to recall qualitative consumption insights such as information on how much water they had consumed in the past week, how much water they consumed daily on average for showering, which use within the home requires the most water, or what source of water had the best quality. This further validated the need for a system that could help with the collection of more precise quantitative information from the household.

Additionally, during the field deployment, we were in constant communication with participants. All of the data was being monitored in real time remotely on a daily basis. This allowed us to validate critical data points, such as the ones showed in all sub-figures of Figure 3. e.g. When we noticed large drops in TDS concentrations, we would corroborate our hypothesis for the change with participants. For example, asking if the rain storage container had collected rain in the past day or asking if they had had power issues during the last few days when the WiFi blackout occurred.

### Human subject protocol compliance

To ensure compliance with ethical guidelines for research involving human subjects, the experimental setup and interview questions underwent review and approval by our institution’s human subject experiment committee (MIT Committee on the Use of Humans as Experimental Subjects). Informed consent was obtained from the study participants, and we strictly adhered to the data handling and privacy regulations outlined in the Declaration of Helsinki.

### Supplementary Information

Supplementary Information.

## Data Availability

All data used to evaluate the system and reach conclusions of the paper is available through *figshare* under the same name as the publication: *Sensor platform for assessment of water usage patterns in informal settlements*.

## Code Availability

All code and building files for system are available at https://github.com/AndresRicoM/axol.
