# Supplementary material for: Sensor platform for assessment of water usage patterns in informal settlements
Source: Sci Rep. 2023 Nov 2;13:18958. doi: 10.1038/s41598-023-46236-3 (PMC10622545; doi:10.1038/s41598-023-46236-3)
Supplement: Supplementary file 1 — Supplementary Information. [file 41598_2023_46236_MOESM1_ESM.pdf]

Supplementary Material

Andres Rico[1,*], Kent Larson[1], and Mayra Gamboa[2]

[1]Massachusetts Institute of Technology, Media Lab, Cambridge, 02139, USA
[2]Universidad de Guadalajara, CS Lab, Guadalajara, 45180, Mexico
[*]aricom@mit.edu

Electronic Design and Hardware Replication

The PCB cases and PCB designs can all be replicated through digital manufacturing techniques.

All code, building files, and material lists for replicating the system are available at: https://github.com/AndresRicoM/axol.

Sensor Manufacturing

PCB Manufacturing

The system's four module's PCB boards were specifically designed for the task presented in the manuscript. All sensors were first manufactured by milling copper plates. Components were manually soldered onto the boards. After testing copper based boards for any errors in circuit designs, we had PCB's professionally manufactured with advanced etching techniques. Components were then soldered manually. After soldering, all of the boards were tested for errors and malfunctions prior to installation in Mexico.

Case Manufacturing

All of the cases and mounts for the sensors were designed specifically for this project. A key challenge for the devices was to properly design water resistance. All of the devices have a main case that is 3D printed. We use a Prusa i3 MK3 printer to manufacture all cases with PLA as our material of choice. Cases then have an acrylic lid that is fastened with screws to avoid water filtering into the system. During the real-life deployment, tape was added on the interface line of the PLA and the acrylic as an extra layer of protection for the boards.

Buoys contain multiple case parts. As 3D printed PLA is not water proof, all of the parts are covered with a water proof resin prior to installation. Once covered, the parts are glued together with waterproof and food safe resins. All of the cases were manufactured within the lab's facilities. Its important to note that scaling of the system could be greatly accelerated through molding, casting and plastic injection manufacturing techniques.

Sensor Installation

Tank Sensor

The tank sensor is placed on the lid of a container. The flattest and most centered spot on the lid is chosen as an ideal location for the sensor. Some lids have tilt that will result in the sensor's accuracy decreasing. The sensor is adhered to the lid by using 3M™ VHB™ two sided adhesive patches. Before adhesion, the lid is cleaned with ethanol so as to avoid having any dust particles that will compromise adhesion. The adhesive is heated up with a lighter so as to vulcanize it to the containers lid. Once in place, we apply pressure with our hands for 20 seconds to secure adhesion and fit.

Bucket Sensor

The bucket sensor is placed on the side of a given bucket. Adhesion is carried out similar to the process described above for the *tank sensor*. The bucket's case has a curvature that allows the adhesive to increase the surface area that is in direct contact with the adhesive and the bucket. This enables devices to have good adhesion independent of bucket curvatures. Correct placement of the bucket sensor is critical for the device to detect bucket usage. For buckets with handles, we place the sensor on one of the two sides of the bucket that have parallel planes to the handle.

Quality Sensor

The quality sensor is left floating inside of the container once it has been assembled. The case has a lower cavity that is filled up with water to balance the center of gravity for the device. We first fill up this cavity with water using a syringe. We then seal the cavity with a rubber plug. You may reference **a** in Figure 1 in the manuscript to see the red plug and the cavity. We then tie a string line to the sensor that will allow us or the community to retrieve the device even when water is low on a given container. This line is attached to an external point of the container so that it is easily accessible.

Battery Life

A critical component for the success of the device is battery life. Longer battery lives, would reduce the mental burden on communities that having to charge or swap batteries can represent. This is also crucial for being able to gain insight into long-term behavioral patterns and statistics. The current implementation's off the shelve low-power electronics, low-power communication protocol (esp-now) and low data collection rates allow for extended battery life on the wireless sensing nodes. Our calculations show that, within lab conditions, a wireless sensor node with a $420mAh$ battery is able to sample at a frequency of two times per day for more than 12 months. Field deployments show that humidity and temperature conditions make it more challenging for theoretical and laboratory experiments to hold. Improvements on case design and the use of higher capacity batteries could greatly extend battery life in the field.

Database Architecture

The HomeHub sends information to a remote server with a PostgreSQL-based database. The database has a relational architecture. This architecture allows for a easy access to all of the information from sensors that are connected to a specific HomeHub. this allows for complete and direct queries when further analyzing the data. The database uses the HomeHub's Mac Address as a primary key for registering any device. This means that a sensor that is not registered to a specific HomeHub will not be allowed to upload information into the database. This helps to ensure that all sensors that are queried for the manuscript's results analysis are registered under the HomeHub that is installed at the specific location of interest. It is important to note that all information is securely stored and anonymized so as to preserve privacy of our study participants.

Structured Interview Questions

This survey questions can help researchers to inform sensor deployments and to better understand water infrastructure of a given household. For this publication, this survey was used to dimension the system, detect the key tanks to place sensors and determine some of the expected behaviors that could be interpreted from the data. This survey can be used as a template for any team that is interested in activating their own sensor system within real-life deployment scenarios. The survey has five different section. Each one focuses on a different aspect of the infrastructure. Note that the section that addresses qualitative information like costs and water consumption is prone to yield answers that might not be very accurate or biased. Those questions can be better addressed and complemented through the sensing system. We note that these survey was used in Mexico, cultural adaptation might be required if it is used in other regions around the world.

Section 1

- On which street or avenue is your house located?
- Between which streets?
- How many people live in your household?
- How many bathrooms are there?
- How many bedrooms are there?

Section 2

- Do you have water in your home?
- How do you receive water in your home?
  a. From a faucet or hose inside my house
  b. From a faucet or hose in my yard or garden
  c. I don't have piped water

  Other:
- Where does the water you use in your home come from?
- Of the following activities, which ones do you do to have water in your home?
  a. Fetching water from a well
  b. Carrying water from a community tap

c. Fetching water from another dwelling dwelling
d. Carrying water from a river, stream, or lake
e. Buy a water pipe
f. Capturing rainwater

Other:

- If you are connected to the public network, how do you rate this service?

Section 3

- Select all the things your home has: Check all that apply.
  a. Tinaco / Tank (open option for capacity)
  b. Cistern (open option for capacity)
  c. Water pump
  d. Shower
  e. Boiler or water heater (gas, electric or firewood)
  f. Water heater (solar)
  g. Washing machine
  h. Toilet (toilet or toilet)
  i. Latrine (well or hole)
- How does your bathroom work?
  a. It has direct discharge of water
  b. I pour water in it with a bucket
  c. You cannot pour water in it
- How does the drainage (sanitation system) or drain of your house work?
  a. You are connected to the public network
  b. It is connected to a septic tank or septic tank (biodigestor)
  c. It is connected to a pipe that will dump outside of the house
  d. It is connected to a pipe that dump to a river, lake, or sea.
  e. I don't have drainage
- When you have to buy water pipes, how long does a pipe last in the dry season? (Specify pipe capacity)
- When you have to buy water pipes, how long does a pipe last in summer? (Specify pipe capacity)
- When you have to buy water pipes, how long does a pipe last in winter? (Specify pipe capacity)
- On average, how many pipes do you buy per year?
- Tell us a little about how you usually use water. Select all the actions that match how you use water in your home.
  a. I collect the cold water from the shower and use it for something else
  b. I water my plants with a hose
  c. I water my plants in the morning or evening
  d. I place dishes under the pots so that the water does not drain out
  e. I collect the water with which I wash dishes or clothes and use it for something more
  f. I reuse the water where I sow vegetables
  g. I leave the shower open while I soap
  h. I turn off the faucet while brushing my teeth

Other:

- How do you use and reuse water?

Section 4

- How much do you pay SIAPA per month for water service?

  a. 0 pesos
  b. from 1 to 150 pesos
  c. from 151 to 300 pesos
  d. from 301 to 450 pesos
  e. more than 451 pesos

- How much does a water pipe cost on average?
- How much did a water pipe cost when you moved to this colony?
- How much is the most you have paid for a water pipe?
- Select the option that best describes your situation when buying water from a pipe: (select only one option)

  a. I always buy a complete pipe for my house
  b. Sometimes I share a pipe with a neighbor
  c. I usually share a pipe with other neighbors
  d. I always share a pipe with other neighbors

- If we add up what everyone living in their household earns in a year, how much of that is spent on paying for the water they consume in that year?

  a. more than half
  b. between half and one-quarter
  c. between one-quarter and 10
  d. less than 10

- Do you think you have a lot to pay for the water you consume?

  a. Yes
  b. No

Section 5

- Would you be interested in learning new techniques to improve water availability in your home?

  a. Yes
  b. No

- Do you currently use any water-saving techniques in your home?

  a. Yes
  b. No

- Would you be willing to make modifications to your current water installation?

  a. Yes
  b. No

- What would be the main reason for making these changes?

  a. Save money
  b. Save water
  c. Facilitating my access to water

- What would be a reason for you not to make these changes?
    a. Lack of money
    b. Lack of someone to help me install it
    c. Lack of interest
- What do you consider to be the biggest challenge you face regarding the issue of water?
- How do you think this challenge could be solved?
